# Human papillomavirus infection: an Anonymous Prevalence Study in South Wales, UK

**DOI:** 10.1038/sj.bjc.6603245

**Published:** 2006-07-04

**Authors:** S Hibbitts, G C Rieck, K Hart, N G Powell, R Beukenholdt, N Dallimore, J McRea, A Hauke, A Tristram, A N Fiander

**Affiliations:** 1 Department of Obstetrics & Gynaecology, Wales College of Medicine, Cardiff University, Heath Park, Cardiff CF14 4XN, UK; 2 County Durham, Darlington Acute Hospital, NHS, Darlington Memorial Hospital, Hollyhurst Road, Darlington DL3 6HX, UK; 3 Department of Pathology, Royal Gwent Hospital, Newport NP20 2UB, UK; 4 Cytology Department, Llandough Hospital, Cardiff and Vale NHS Trust, Cardiff CF64 2XX, UK; 5 Cervical Screening Wales, 18 Cathedral Rd, Cardiff CF11 9LH, UK

**Keywords:** HPV prevalence, cervical cancer, HPV vaccine

## Abstract

The objective of this study was to describe human papillomavirus (HPV) prevalence in South Wales in relation to age, cytology and social deprivation. This was an unlinked, prospective, anonymous, population-based study. DNA was purified from 1911 liquid-based cytology samples (mean age 37.7 years, cytology 93.2% negative, social deprivation average score 17.9) using quality assured techniques and the presence of virus determined by PCR-Enzyme Immuno Assay (PCR-EIA). 209 (10.9%) samples contained high-risk (HR) HPV infection of which 36.4% had multiple HR-HPV types. The most frequent HR types were HPV 16 (19.6%), HPV 35 (9.5%), HPV 66 (9.2%), HPV 59 (8.5%) and HPV 56 (7.6%). There was a strong association between HPV infection and cytological abnormality. Significantly more HR-HPV infections were detected in women under the age of 30 years (68.9% of all HR-HPV infections Fisher's exact test *P*=0.0001) compared to 30 years and above. There was no difference in HPV prevalence between different socioeconomic groups. The data presented suggest a different HPV type distribution in South Wales in comparison to that reported for other populations.

Cervical cancer is the second most prevalent female cancer worldwide (Parkin *et al*, 2005). HR-human papillomavirus (HPV) infection plays a central role in cervical carcinogenesis, with HPV DNA identified in 99.7% of invasive cervical carcinomas (Walboomers *et al*, 1999). However, HPV infection is common and in the majority of cases self-limiting. An estimated 70% of infections are transient and cleared within 18 months, with less than 1–2% of high-risk infections resulting in cervical cancer. There is a large discordance between the number of women infected (up to 80% cumulative lifetime risk), and those who develop cervical cancer. Further research is required to define prognostic biomarkers that will identify women in whom HPV infection is likely to progress to cervical neoplasia. Factors that may influence the outcome of infection include HPV type, viral load and integration status.

The prevalence of HPV- and HPV-type-specific distribution varies between different geographical regions worldwide (Clifford *et al*, 2005a). HPV 16 and 18 are associated with an increased risk of progression to cervical neoplasia (Clifford *et al*, 2003, 2005b). However, the incidence of different HPV types in cervical cancers has been found to vary with different geographical populations (Bosch *et al*, 1995). Only a limited number of studies have investigated HPV prevalence within the UK (Cuschieri *et al*, 2004; Peto *et al*, 2004) with the results of the largest study to date expected in November 2008 (Smyth *et al*, 2004).

HPV prevalence is age dependent with a peak in women below the age of 25 (Schiffman *et al*, 1993; Petignat *et al*, 2005). In some populations, however, a second peak in women over 55 has been observed (Herrero *et al*, 2000; Molano *et al*, 2002). Social deprivation has also been associated with cervical cancer incidence, but the link is likely to be complex, as other factors such as smoking, education and reduced participation in screening must be considered (Sweetnam *et al*, 1981; Murphy *et al*, 1990; de Sanjose *et al*, 1997; Krieger *et al*, 1999; Thomas *et al*, 2001; Parikh *et al*, 2003; McFadden *et al*, 2004). However, not all studies have been able to confirm this correlation (Parazzini *et al*, 1998).

Prophylactic HPV vaccines are currently in phase III trials and should be available within 5 years. Prevalence studies are required to identify baseline data against which efficacy of vaccination can be compared, elucidate if type-replacement occurs following vaccination and determine what fraction of disease is prevented. Current vaccines only protect against HPV16 and 18 (Harper *et al*, 2004; Villa *et al*, 2005). Our laboratory has demonstrated that other HPV types are linked with anogenital neoplasia in South Wales (KW Hart, personal communication), underlining the need to identify type-specific prevalence in this region.

Determination of HPV prevalence requires a nonselected population, such as prospective sampling of screening groups, although this will not capture those who fail to attend for screening. When coverage by the screening programme is high, sampling is likely to reflect the true prevalence of HPV infection within a population. Studies of HPV infection with informed consent may result in bias, as women may refuse testing or be excluded for logistical reasons. For these reasons prospective anonymous sample collection was chosen to investigate HPV prevalence within South Wales.

Here, we describe the first HPV prevalence study in Wales and assess if there is an association between social deprivation, age or cytology and HPV type.

## MATERIALS AND METHODS

### Clinical cohort

In total, 2023 consecutive screening samples were collected, with the assistance of Cervical Screening Wales (CSW), over a 5-month period in 2004. Cervical Screening Wales manages an organised call–recall system inviting women aged 20–65 years for 3 yearly cervical screening. The residual material of liquid-based cytology (LBC) samples from women attending for routine cervical screening were used for this study. Inadequate cytology samples or those from colposcopy clinics were excluded. This study was approved by South East Wales Local Research Ethics Committee.

### Sample processing

The LBC samples (Thinprep, Cytyc Corp, Boxborough, MA, USA) were processed and analysed by the Cytology Laboratory at Llandough Hospital, Cardiff, Wales. The residual specimen was anonymised and transported to the HPV Laboratory, Wales College of Medicine, Cardiff University. Samples were centrifuged, washed with 10 mM Tris-HCL (pH 7.4) and resuspended in 1 ml 10 mM Tris-HCL (pH 7.4) and stored at −80°C until required for further analysis.

### DNA purification and PCR-EIA

Of 10 mg ml^−1^ proteinase K (Boehringer Mannheim) 10 *μ*l was added to a 100 *μ*l aliquot from each sample and incubated at 56°C for 2 h, followed by 100°C for 10 min. Samples were allowed to cool, then centrifuged at 13 000 r.p.m. for 10 min, and the supernatant transfered to an appropriately labelled tube.

Standard HPV typing of LBC samples was performed using the PCR-Enzyme Immuno Assay (PCR-EIA) method of Walboomers *et al* (Jacobs *et al*, 1997) with minor modifications. PCR reactions were performed in a final volume of 25 *μ*l and PCR cycling conditions were 94°C – 4 min, then 40 cycles of 94°C – 30 s, 40°C – 90 s, 72°C – 60 s followed by 72°C – 4 min. Positive (CaSki) and negative (water) PCR/ELISA controls were included in every experiment, as were positive and negative DNA extraction controls. A standard PCR for the house-keeping gene *β*-globin was also performed on each sample to ensure PCR viability. 10% of samples analysed were repeated to determine reproducibility.

### Selection criteria for samples included in analysis

Detailed analysis was only performed on samples that conformed to the following criteria:

1. *β*-Globin PCR positive (96% extraction efficiency)
2. Complete information available on age, cytology and social deprivation score
3. Within the target screening age group 20–65 years

This eliminated 112 of the original 2023 samples. Detailed analysis was performed on the remaining 1911 samples.

### Cytology results

Cytology results were reported according to the guidelines of the British Society of Cervical Cytology (BSCC) by the laboratory at Llandough working within the CSW programme.

### Age standardisation

The HR-HPV prevalence was age-standardised using the European Standard Population (Waterhouse, 2003) and the following age-standardised rate calculation: 
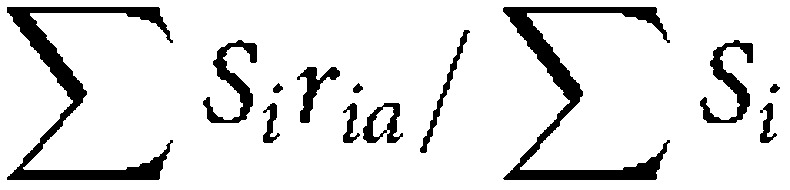
 where *S*_*i*_ is the standard population sizes in the relevant age groups and ‘*i*’ and ‘*r*_*ia*_’ are the age-specific rates in age groups *i* and area *a* (Breslow and Day, 1987).

### Social deprivation score

Social deprivation was estimated by linking postcodes to the Welsh Index of Multiple Deprivation (WIMD) (http://www.wales.gov.uk/keypubstatisticsforwales/wimd2005.htm). WIMD is a Welsh Assembly Government funded project, developed to describe levels of deprivation across Wales and support policy development and the targeting of resources. The index provides an estimate of social deprivation based on income, employment, health, education, housing, access to services and physical environment. Each area within Wales has been ranked according to social deprivation score; rank 1 being the most deprived area with the highest social deprivation score (78.9); the mid-range rank having a score of 17.9 and the lowest rank a social deprivation score of 1.4.

## RESULTS

### Study population

**Figure 1:**
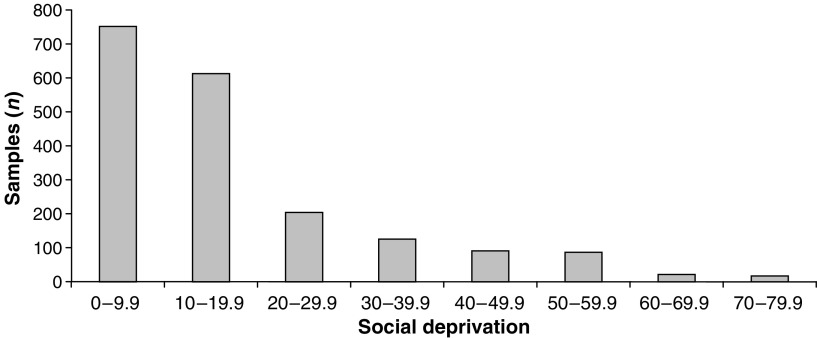
Distribution of samples according to social deprivation score (2005). The number of samples in each social deprivation score grouping from 0–9.9 to 70–79.9 in the total 1911 samples analysed.

Of the 1911 samples included in the analysis, the mean age was 37. 7 years with 31.3% aged between 20–29 years, 26.4% 30–39 years, 22.2% 40–49 years, 14.9% 50–59 years and 5.2% over 60 years old. In all, 93% were cytology negative, 3.9% had borderline changes and 1.7, 0.4 and 0.7% had mild, moderate and severe dyskaryosis, respectively. Figure 1 illustrates the distribution of samples according to social deprivation score with 39.4% between 0–9.9, 32% 10–19.9, 10.8% 20–29.9, 6.6% 30–39.9, 4.7% 40–49.9, 4.6% 50–59.9 and 1.9% over 60. Thus, the majority of our sample population is from areas not suffering notable deprivation.

### HPV typing

317 HR-HPV infections were identified in 209 samples and an additional 83 LR-HPV infections found. Of the 209 HR-HPV positive samples, 114 were single infections, 59 were multiple infections of HR types and 36 were multiple infections with both LR and HR-HPV. There were 47 samples with single LR-HPV infections. In total, 256 HPV infected samples were identified in the 1911 samples included in the analysis, giving an overall HPV prevalence for either HR or LR HPV of 13.4% (95% CI 11.9–15%). The age standardised HR-HPV prevalence in the Welsh population was 14.6%.

**Figure 2:**
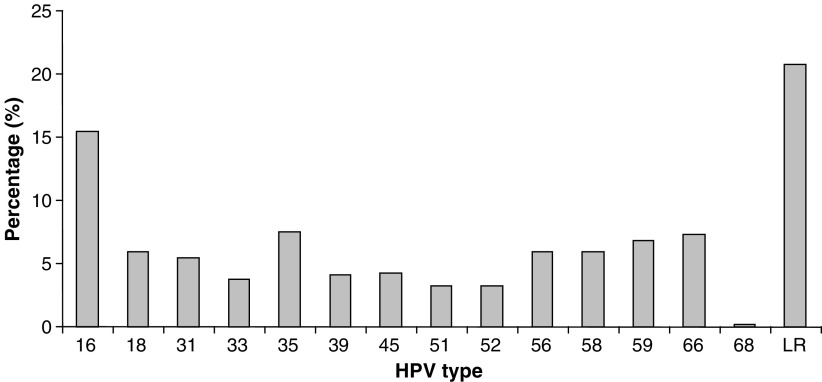
HPV type distribution in the Welsh population. The distribution of HR-HPV types and LR-HPV as a percentage of the total number of HPV infections observed in this sample set (*n*=400).

The distribution of HPV types is illustrated in Figure 2. The distribution of specific HR-HPV types (*n*=317) was as follows: HPV 16 (19.6%), HPV 35 (9.5%), HPV 66 (9.2%), HPV 59 (8.5%), HPV 56 (7.6%), HPV 58 (7.6%), HPV 18 (7.6%), HPV 31 (6.9%), HPV 45 (5.4%), HPV 39 (5.1%), HPV 33 (4.7%), HPV 52 (4.1%), HPV 51 (4.1%) and HPV 68 (0.3%).

Repeat experiments to validate the reproducibility of our results identified 96% consistency in HR-HPV detection and 92% in HR-HPV typing.

### HPV prevalence and cytology

**Figure 3:**
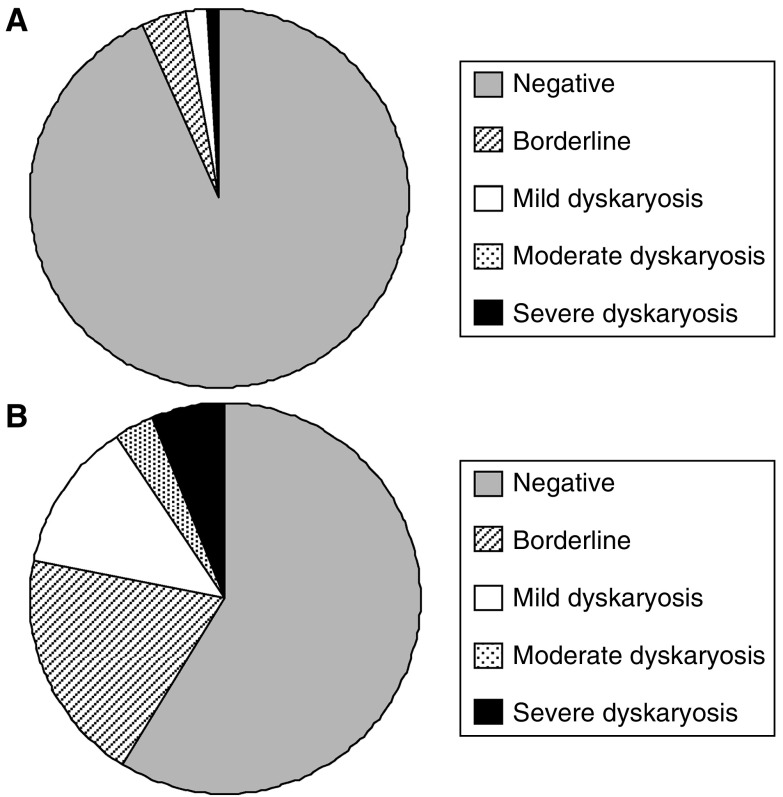
Comparison of Cytological Grades. (**A**) The number of samples in each cytology grade as a percentage of the total 1911 samples analysed. (**B**) The number of HR-HPV samples in each cytology grade as a percentage of the 209 HR-HPV samples identified.

**Figure 4:**
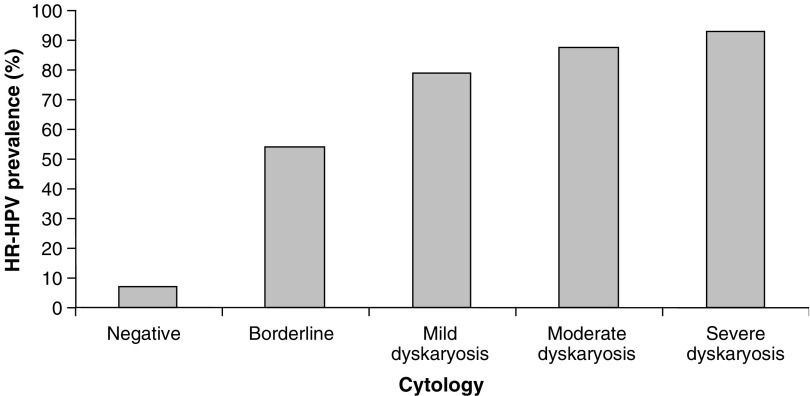
Percentage of HR-HPV positive in each cytology grade. The percentage ratio of HR-HPV samples to the total number of samples identified in each cytology grade.

**Figure 5:**
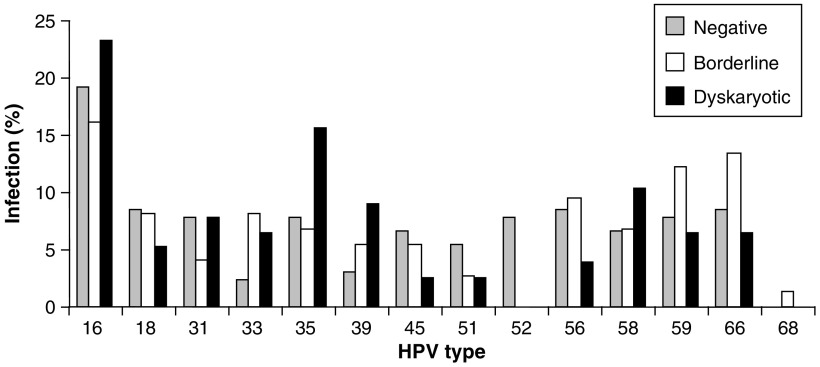
HPV-type-specific distribution and cytology grade. The number of each HR-HPV type differentiated according to the cytology grades: negative, borderline and dyskaryotic (mild, moderate and severe), calculated as a percentage of the total number of HR-HPV infections in each of these cytology grade groups.

Comparison of cytological grades in the whole data set (*n*=1911) with those in the HR-HPV positive samples (*n*=209) (Figure 3A and B, respectively) reveals a significant decrease in the number of cytology negative samples (93–59%) and an increase in borderline, mild, moderate and severe cytology cases in the HR-HPV positive samples. There was a marked increase in HPV prevalence with the degree of dyskarosis detected from 6.9% in cytology negative samples up to 92.9% in samples with severe dyskaryosis (Figure 4). A breakdown of the incidence of specific HPV types in relation to cytological grade (Figure 5) identifies HPV 16, HPV 35, HPV 39 and HPV 58 as more prevalent in dyskaryotic cells, with HPV 39 showing significantly higher incidence in dyskarotic than negative samples (Fisher's exact test *P*=0.0112).

Negative and borderline cytology samples had a similar percentage of single (4 and 23%, respectively) and multiple (2.2 and 25.7%, respectively) HR-HPV infections. In dyskaryotic samples multiple HR-HPV infections were more prevalent in mild (42.4%) than in those with a moderate (12.5%) and severe cytological grading (21.4%), whereas single HR-HPV infections were predominant in the moderate (75%) and severe (57.1%) dyskarotic samples compared with those observed in the mild (33.3%).

### HPV prevalence and age

**Figure 6:**
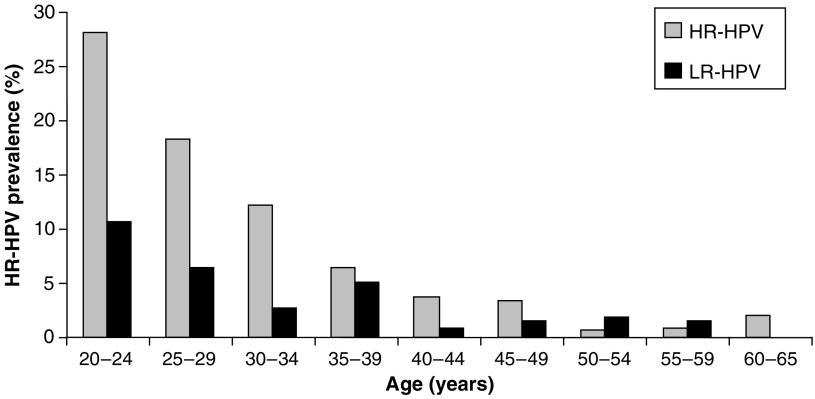
Percentage of women in each age group infected with HR and LR-HPV. The percentage ratio of HR-HPV and LR-HPV samples to the total number of samples identified in each age group.

In all, 68.9% of HR-HPV and 64% of LR-HPV infections were found in women aged under 30 years of age. The percentage of HR-HPV infections observed within each age group decreased progressively from 28% among 20–24 year olds, to 3% in those aged 45–49 years with between 0.6 and 2% positive in women aged 50 and 65 years (Figure 6). 75% of multiple HPV infections observed were in women under 30 years old. Women aged between 50 and 59 years had a higher percentage of LR-HPV infections than HR-HPV infections with 1.9 and 1.6% LR-HPV in women 50–54 years and 55–59 years, respectively compared to 0.6 and 0.8% HR-HPV although not statistically significant (Fisher's exact test *P*=0.4000).

### HPV prevalence and social deprivation score

**Figure 7:**
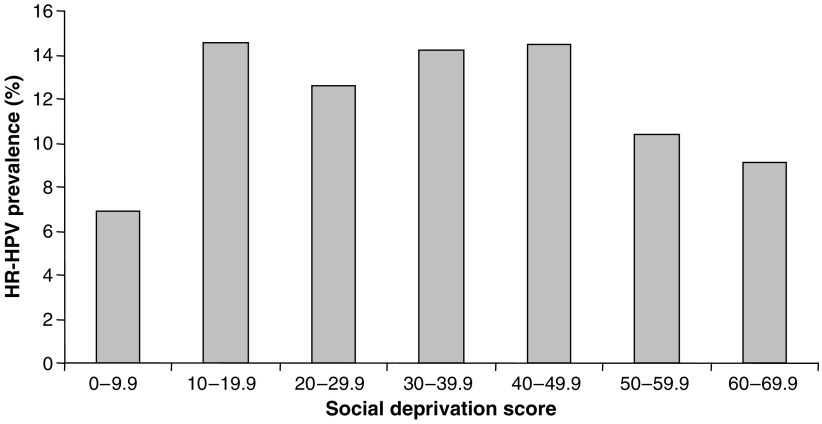
Percentage of women in each social deprivation score group infected with HR-HPV. The percentage ratio of HR-HPV samples to the total number of samples identified in each social deprivation score group.

No correlation between HR-HPV prevalence and social deprivation score was observed (Figure 7). The percentage of women with HR-HPV infections when grouped according to social deprivation score was between 7 and 15%.

## DISCUSSION

### Overview

The use of anonymous consecutive routine screening samples in this study limits population bias with no women excluded. Collection of the samples over a period of less than 6 months avoided testing of the same subjects twice due to recall for repeat smears. The Welsh population represents an ideal study cohort since it is demographically stable and socioeconomically well-defined.

### HPV prevalence

Variability between HPV prevalence in different populations has been highlighted in numerous studies but is complicated by differences in study design, sample collection and methods used for HPV detection and typing. A recent publication by Clifford *et al* (2005a) reported data generated from the general populations of 13 areas from 11 countries and identified large differences in HPV prevalence in cytologically normal women ranging from 1.4% in Spain up to 25.6% in Nigeria. There are currently relatively few published HPV prevalence studies from the UK (Cuschieri *et al*, 2004; Peto *et al*, 2004) and even fewer reporting age-standardised prevalence. In this cohort, we found an overall HPV prevalence of 13.4% with an age standardised HR-HPV prevalence of 14.6%. The 95% confidence interval of 11.9–15% indicates that this is likely to be a reasonably accurate representation of HPV prevalence within the 206 369 women tested per annum by CSW from the Welsh population. A direct comparison of this data with that obtained from a Scottish cohort (Cuschieri *et al*, 2004) illustrates an overall lower HPV prevalence in Wales than in Scotland (20.5% overall and 15.17% HR-HPV in Scotland (not age-standardised)). However, within a European context, the HPV prevalence in Wales would still be considered high, relative to prevalences of 8.8% in Turin, Italy (Ronco *et al*, 2005) and 3.0% in Barcelona, Spain (de Sanjose *et al*, 2003).

HPV 16, HPV 35, HPV 66 and HPV 59 were the most prevalent HPV types in South Wales. However, comparison of our data with the Scottish cohort identified an overall lower percentage of HPV 16, HPV 18, HPV 31, HPV 51, HPV 52 and HPV 68 and an increase in HPV 35, HPV 66 and LR-HPV types. The high incidence of HPV 35 has been reported in other papers using the PCR-EIA method of Walboomers *et al.* (Wall *et al*, 2005) and future work will investigate this further.

### HPV prevalence and cytology

Comparison of HR-HPV prevalence in relation to cytological grade showed a strong association between HPV infection and cytological abnormality (Figure 4). We also compared the distribution of HR types among negative cytology samples with the distribution in borderline and dyskaryotic samples (Figure 5); this indicated that HPV 16, HPV 35, HPV 39 and HPV 58 were all overrepresented in dyskaryotic cytology samples relative to their prevalence in borderline and negative cases and, in the case of HPV 39, this was statistically significant. This raises the interesting possibility that some types may be more strongly associated with cellular dyskaryosis than others as previous studies have highlighted (Munoz *et al*, 2003). It will be interesting to see whether such associations mirror the association of certain types (notably HPV 16) with invasive disease. To gain a full understanding of the relationships between HPV type, dyskaryosis and carcinoma, further studies are required including an assessment of the specific HPV types found in invasive carcinomas in the Welsh population.

### HPV prevalence and age

HPV prevalence was highest in women aged 20–30 years. This is consistent with other studies and is likely to be associated with a higher number of recent sexual partners among this age group. HR-HPV types were detected more frequently than LR-HPV types in women aged 20–49 years and in all dyskaryotic cytology grades. However, as reported in previous studies (Chan *et al*, 2002) a slight increase in LR-HPV over HR-HPV was observed in women over 50 years old.

### HPV prevalence and social deprivation score

There was no correlation between HR-HPV and social deprivation score. However, it must be borne in mind that this study does not provide a complete picture of social deprivation related to HPV infection. This is because although use of a screening population avoids many potential sources of bias, it inevitably only includes people who attend for their smears. Hence, we do not have data on the HPV status of nonattendees, and as this group has a higher average social deprivation score than those who do attend for smears, we cannot conclusively say that a correlation between HPV infection and social deprivation does not exist (average social deprivation score for women in this study=17.9, average social deprivation score for women not attending for smears in 2004=21.9 indicating that nonattenders tend to reside in areas of higher social deprivation (unpaired *t*-test *P*<0.0001)). It would be interesting to study HPV infection in screening nonattendees but such a study would be logistically problematic, although possible using self-testing (Nobbenhuis *et al*, 2002).

### HPV vaccines and the future

With prophylactic vaccination against HPV imminent, there is an urgent need for accurate epidemiological studies to provide baseline data, and so inform vaccine design regarding inclusion of cohort-specific HPV types. Given the varying oncogenic potential of different HPV types (Munoz *et al*, 2004), HPV type prevalence is not the only factor influencing vaccine composition, nonetheless, it is a significant factor. The data presented here suggests that a vaccine purely based on HPV 16 and 18 may be less effective in reducing cervical cancer incidence in the Welsh population than in others.

## CONCLUSION

In conclusion, HPV prevalence in South Wales is high, with HPV 16 and HPV 35 recognised as the two predominant circulating HR-HPV types. Future work will aim to investigate a larger sample set (*n*=10 000) to confirm HPV prevalence and the predominant HPV types within South Wales.
